# Characterization of redox and salinity-tolerant alkaline protease from *Bacillus halotolerans* strain DS5

**DOI:** 10.3389/fmicb.2022.935072

**Published:** 2022-08-18

**Authors:** Yangxuan Wen, Jiyu Qiang, Guixu Zhou, Xiaobo Zhang, Lei Wang, Yawei Shi

**Affiliations:** Key Laboratory of Chemical Biology and Molecular Engineering of Ministry of Education, Institute of Biotechnology, Shanxi University, Taiyuan, China

**Keywords:** alkaline protease, identification, purification, enzymatic properties, *Bacillus halotolerans*

## Abstract

Bacillu*s halotolerans* DS5 was isolated and identified as a halophilic microbe according to 16S rRNA analysis and the physical and chemical indices of the strain. A new alkaline protease (designated as prot DS5) from *Bacillus halotolerans* DS5 was produced, purified, and characterized. After 12 h incubation in the medium with 1% dextrin, 0.5% NaCl, 2% soluble starch, and 1% yeast extract (pH 7.0), it could reach the maximum enzyme activity (279.74 U/ml). The prot DS5 was stable in the pH range of 6.0–12.0 and the temperature range of 40–60°C, with maximal hydrolytic activities at pH 9 and at 50°C. In the presence of Ca^2+^, Mn^2+^, Ba^2+^, Mg^2+^, and Fe^3+^, protease activity was enhanced. The prot DS5 was maintained highly stable in NaCl (up to 2.5 mol/L), reducing and oxidizing agents. The prot DS5 also exhibited compatibility in other detergent ingredients, such as non-ionic and anionic surfactants. These properties of prot DS5 make this enzyme suitable for various industrial applications (e.g., detergents and leather).

## HIGHLIGHTS

- *Bacillus halotolerans* DS5 produces extracellular alkaline protease (prot DS5) and achieves the highest activity after 12 h incubation.

- Prot DS5 is a manganese ion-activated protease and exhibits stability to salt, reducing agents, and oxidizing agents.

- As a novel biological additive, prot DS5 has great potential in the detergent industry.

## Introduction

Protease, an enzyme that catalyzes the hydrolysis of proteins, has become one of the most widely used enzyme preparations in the world (Nandan and Nampoothiri, 2020). It has an enormous range of applications in detergents, leather, textiles, dairy products, food, feather processing, and other industrial fields, as well as in the biopharmaceutical-cosmetics industry (Mechri et al., 2017). According to the position of the protease action site in the peptide chain, it can be preliminarily divided into exopeptidase and endopeptidase, and based on the catalytic mechanism, endopeptidases are divided into four subclasses, namely, serine proteases, aspartic proteases, cysteine proteases, and metalloproteases (Mothe and Sultanpuram, 2016). According to the optimum reaction pH of protease, it is divided into alkaline, neutral, and acidic proteases (Maruthiah et al., 2013). Alkaline protease is an enzyme that hydrolyzes protein peptide bonds under the condition of alkaline pH, and its optimum pH value is generally 9–11 (Mirzapour Kouhdasht et al., 2018). Many bacterial alkaline proteases used as detergents are commercially available, such as subtilisin Carlsberg, savinase, and subtilisin BPN (Gupta et al., 2002).

Microorganisms have become the main source of enzymes due to their advantages, such as simple culture methods, wide sources, and rapid reproduction. At present, *Bacillus* is a kind of bacteria that is widely used in microbial resources, because it can produce resistant spores and has strong survival adaptability. It has been widely used in agriculture, animal husbandry, industry, medicine, environmental protection, and other fields. To date, the main method for obtaining stable proteins is to screen microorganisms from extreme environmental conditions (Yang et al., 2014). Salt-tolerant bacteria, also known as facultative halophilic bacteria, can grow and reproduce in both salt and salt-free environments. They are a rich source of salt-tolerance genes and are also a resource guarantee for the study of the physiological mechanism of salt-tolerance and the isolation and application of salt-tolerance genes. The large-scale application of enzymes is limited by harsh industrial environments, such as high salt, high temperature, and strong acid/alkali. The enzymes produced by halophilic bacteria can maintain high activity and stability under high-salt conditions, so they have extremely high application potential (Karray et al., 2021).

The old name for *Bacillus halotolerans* is *Brevibacterium halotolerans* (Ben-Gad and Gerchman, 2017). *Bacillus halotolerans* is a rhizosphere-promoting bacterium with the ability to promote plant growth and enhance plant resistance to drought and salinity stress (Zhang et al., 2018). *Bacillus halophila* QTH8 isolated from the Cotinus coggygria rhizosphere soil against wheat crown rot (Li et al., 2022). *Bacillus halotolerans* strain MS50-18A was performed by Jaime Sagredo-Beltrán and co-workers (Sagredo-Beltrán et al., 2018), followed by the analysis of antifungal activity against root rot causal phytopathogens. Recently, a serine alkaline protease from *Bacillus halotolerans* CT2 (Dorra et al., 2018) has been purified and characterized. To date, alkaline proteases from *Bacillus halotolerans* have been characterized or explored for potential industrial applications, but the production of commercial enzymes from them was totally unsighted. The fermentation cycle, as well as the relatively low stability and catalytic activity under relevant operating conditions (i.e., high temperature, high pH, and high salt), often hinders the widespread application of enzymes from *Bacillus halotolerans*.

During the last few years, many extreme enzymes have been studied, including thermophilic (Dai et al., 2022), psychrophilic (Santiago et al., 2016), acidophilic (Parashar and Satyanarayana, 2018), alkalophilic (Ito et al., 1998), halophilic (Mokashe et al., 2018), radiophilic, and barophilic enzymes (Raddadi et al., 2015), but the number of extreme enzymes is currently insufficient to meet growing industrial demands (Mesbah, 2022). Halophilic microorganisms have natural advantages in protease production, and many new extreme enzymes can be developed.

In this context, we obtained a novel alkaline protease named prot DS5 from *Bacillus halotolerans* with fast growth rate and short fermentation period. Then, the main properties of protease including temperature and pH optima and stability, metal ion, salt, reducing agents, oxidizing agents, and surfactants have been determined. Moreover, the application of extracellular proteases produced by the isolate *Bacillus halotolerans* DS5 as a detergent additive in the washing industry was evaluated.

## Materials and methods

### Materials

The soil samples used for screening are from the vegetation surface rich in withered grass leaves in the mountainous area of Zuoquan, Shanxi Province, China (37°07′ north latitude, 113°37′ east longitude). Dextrin, casein, soluble starch, and borax were purchased from Sinopharm Reagent. Other reagents are analytical pure.

### Screening of the protease-producing strain

In the preliminary screening, the soil samples are dispersed in a sterile petri dish and then placed at about 80°C for 24 h to kill most heat-labile bacteria. Then, 1.0 g of the heat-treated sample was weighed into 10 ml of sterile water to make a soil suspension, which was then serially diluted with sterile water to make six concentration gradients ranging from 10^–1^ to 10^–6^, with each dilution 100 μl of the suspension was evenly spread on the milk plate. After incubating for 12 h at 37°C, a clear transparent circle is formed around the colony-producing protease, and the diameter of the transparent circle/colony diameter (D/d) is measured.

The strains obtained from the preliminary screening were inoculated into the liquid enzyme-production medium and cultured in a constant temperature shaker at 37°C for 12 h. The medium was then centrifuged, and the activity of the supernatant was measured. The microbial strains with high enzyme activity were screened for subsequent analysis.

### Identification of the protease-producing strain

The protease-producing strains were identified according to their biochemical and physiological characteristics; morphological, including gram staining, shape, motility, production of catalase, amylase, and hydrogen sulfide; carbohydrate fermentation patterns; and the growth ability at different salt concentrations (Zhao et al., 2016). The PCR amplification template was the genomic DNA. Universal primers for bacterial 16S rRNA amplification were 5′-AGAGTTTGATCCTGGCTCAG-3′ and 5′-TACCTTGTTACGACTT-3′. PCR program was 95°C for 30 s denaturation, 55°C for 30 s annealing, 72°C for 10 min extension, and 30 cycles (Hu et al., 2013). Its products were recovered using a Biomiga DNA Gel Purification Miniprep Kit (Biomiga, Hangzhou, China) and sequenced by Sangon Biotech (Shanghai, China). The sequence obtained by sequencing was aligned online using Blast in NCBI, and the neighbor-joining method in MEGA 6.0 was used to build a phylogenetic tree.

### Production and purification of prot DS5

A single *Bacillus halotolerans* DS5 was inoculated into 5 ml LB medium and shaken overnight at 37°C and 200 rpm. The seed liquid was transferred to 300 ml of fermentation medium and fermented under the above conditions for about 24 h. During the incubation, samples were determined the enzyme activity and nucleic acid amount for every 2 h (OD_260_). Cell growth was monitored by the amount of nucleic acid according to reference (Prasetyo et al., 2011).

The secreted prot DS5 in the clear cell-free supernatant was precipitated using 75% (w/v) NH_4_SO_2_ precipitation. The precipitated protease was obtained by centrifugation at 13,000 rpm for 30 min at 4°C, resuspended in 50 mM Tris–HCl, 100 mM NaCl (pH 9.0), and centrifuged again. The supernatant was loaded onto a Sephadex G-25 desalting column equilibrated with the above buffer. Prot DS5 was concentrated using a 10 kDa MWCO membrane and applied onto a Superdex-75 column equilibrated with the same buffer. Each 2 ml fraction was collected and analyzed for the alkaline protease activity. Each step of purification was checked by 12% SDS-PAGE.

### Protein mass spectrometry identification

The MALDI-TOF mass spectra were used to analyze peptide mass fingerprinting and MS/MS ion search. The excised protein bands are enzymolyzed and then put into the mass spectrometer for data collection. Peptidecalibstandard II was used for instrument calibration. The data acquisition mode of the primary mass spectrum adopts positive ion reflection mode and automatic data acquisition mode; 5–10 peaks with better signal intensity were selected from the primary mass spectrum peaks for secondary mass spectrometry analysis. The primary and secondary mass spectrometry data were integrated using the Bio Tools software. The Mascot software was used for mass spectrometry data search. The search database was NCBI, the species was the whole species, the cutting enzyme was pancreatin, and the Mascot score was higher than 55. The identification was considered successful.

### Estimation of enzymatic activity

Protease assays were performed according to methods described by Carrie Cupp-Enyard with minor modifications (Hiraga et al., 2005). Briefly, 500 μl of enzyme solution and 500 μl of casein solution (10 mg/ml) were mixed and reacted for 10 min at 40°C; 1 ml of trichloroacetic acid (TCA; 0.4 mol/L) was added to stop the reactions. The absorbance measures 680 nm. The control was treated the same as above, except TCA needs to be added before the enzyme. Under standard conditions, the amount of enzyme required to produce 1 μg of tyrosine/min was defined as 1 unit of enzyme activity.

### Enzyme characteristics

#### Effects of temperature and pH on prot DS5 activity

To determine the optimal temperature for prot DS5, the enzyme activity was measured at 20–80°C under standard experimental conditions. Prot DS5 was incubated at 50°C for various times (30–150 min) to measure protease activity to determine the stability of the enzyme at 50°C.

The optimum pH for prot DS5 was studied in the range of 4.0–12.0. Casein was prepared in different buffers, including citrate (pH 4.0–5.0), Na_2_HPO_4_-NaH_2_PO_4_ (pH 6.0–7.0), Tris–HCl (pH 8.0–9.0), and glycine-NaOH (pH 10.0–12.0).

The pH stability of prot DS5 was studied by the following. The protease (0.4 mg/ml) was mixed with different buffers (pH 6.0–12.0) and incubated for 2.5 h, and then, the enzyme activity was determined. Untreated prot DS5 from the above study served as a control (100%).

#### Effects of metal ions and chemical reagents on enzyme activity

Prot DS5 was preincubated for 1 h with the following metal ions at a final concentration of 1, 5, 10, and 15 mM, including Ca^2+^, Ba^2+^, Mg^2+^, Mn^2+^, Ni^2+^, Fe^3+^, Fe^2+^, and Cu^2+^, and the enzymatic activity was determined.

To study the influence of surfactants agent on prot DS5, the enzyme was preincubated with different chemical reagents, including dithiothreitol (DTT), phenylmethanesulfonyl fluoride (PMSF), ethylenediaminetetraacetic acid (EDTA), β-mercaptoethanol (β-ME), Tween-20, Tween-80, TritonX-114, TritonX-100, alcohol ethoxylate, hydrogen peroxide, sodium dodecyl sulfate (SDS), and cetyltrimethylammonium bromide (CTAB) for 1 h. Then, the enzymatic activity was determined. Prot DS5 activity determined without any additives was used as a control (100%).

#### Effect of salinity on prot DS5

The salinity stability of the enzyme was studied by preincubating the prot DS5 for 1 h under the 0.1 M, 0.5 M, 1.0 M, 1.5 M, 2.0 M, and 2.5 M NaCl. The absence of NaCl was used as a control.

#### Effect of different substrates on prot DS5

Gelatin, casein, skimmed milk, bovine hemoglobin, and BSA as substrates were prepared at 1% concentration. The enzymes were incubated at 40°C for 10 min in different substrates, and then, the activity was determined.

### Determination of kinetic parameters

The prot DS5 activity from casein with different concentrations of 0.36–4.85 mmol/L was measured at 40°C and pH 10.5. Kinetic parameters, *K*_*m*_ (Michaelis-Menten constant), *K*_*cat*_ (turnover number), *V*_*max*_ (maximal velocity), and *K*_*cat*_/*K*_*m*_ (catalytic efficiency), are determined according to the Lineweaver-Burk plot. [E] is the protease concentration in the reaction mixture used for determining the *K*_*cat*_ value. The calculation formula is as follows: *K*_*cat*_ = *V*_*max*_/[E].

### Decontamination ability analysis

The prot DS5 washing ability test was evaluated according to Jian Zhang et al. (2019). We use the following detergents: a (2 g Chinese national standard detergent), b (2 g Chinese national standard detergent + 1% PB92 alkaline protease), and c (2 g Chinese national standard detergent + 1% prot DS5). The test cloth was JB-02 protein test cloth (3 cm × 3 cm). The detergency of the detergent was evaluated according to the method of GB/T13174-2008 (China National Standard). All the assays were conducted in triplicates.

### Statistical analysis

All the assays were carried out in triplicate, and data are presented as mean ± standard error using Student’s *t*-test.

## Results

### Screening and identification of DS5 strain

In total, 80 strains were screened from soil samples to produce transparent circles on milk plates and then rescreened with fermentation culture. There were 3 strains with higher enzyme activity (Supplementary Table 1), and the DS5 strain that produced the highest protease activity was selected for follow-up research.

**FIGURE 1:**
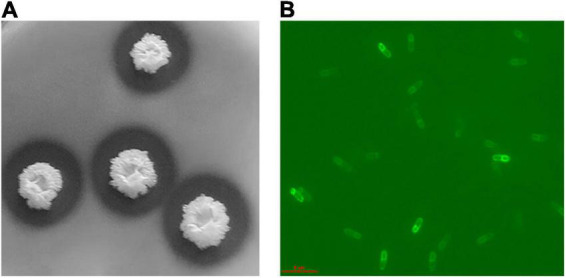
The morphological characteristics of *Bacillus halotolerans* DS5. **(A)** The hydrolyzed transparent circle of *Bacillus halotolerans* DS5; **(B)** Fluorescence microscope image of *Bacillus halotolerans* DS5.

The *Bacillus halotolerans* DS5 was characterized as Gram positive. The cells were short rod-shaped, individually arranged with flagella. The colony was milky white, opaque, rough and wrinkled, and round in shape and exhibits catalase and amylase activities (Figure 1). It was able to grow at 37°C and tolerate up to 10% (w/v) NaCl. In addition, it produced hydrogen sulfide. The fermentation curve showed that it could utilize carbon sources, such as glucose, sucrose, lactose, fructose, maltose, xylose, arabinose, Inositol, sorbitol, mannitol, citrate, and cellulose. The results of its taxonomical characterization are summarized in Table 1.

**TABLE 1:** Identification of the physiological characteristics of *Bacillus halotolerans* DS5.

Items	Results
Gram stain	+
Glucose	+
Sucrose	+
Lactose	+
Maltose	+
Fructose	+
Xylose	+
Arabinose	+
Inositol	+
Sorbitol	+
Mannitol	+
Moveability	+
Salt concentration 10%	+
Salt concentration 20%	−
Catalase activity	+
Cellulose	+
V-P	+
Reduction of nitrate	+
Citrate	+
Hydrogen sulfide production	+
Amylase activity	+

+Indicates that the result is positive, and −Indicates that the result is negative.

**FIGURE 2:**
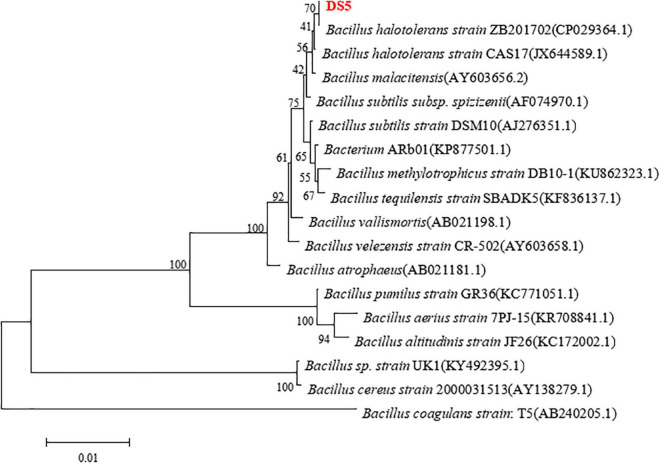
Phylogenetic tree analysis of *Bacillus halotolerans DS5*. Bootstrap values (expressed as percentages of 1,000 replications) are listed at branching points. The scale bar represented 1% divergence. The strains of each genome are as follows: *Bacillus halotolerans* strain ZB201702 (GenBank: CP029364.1), *Bacillus halotolerans* strain CAS17 (GenBank: JX644589.1), *Bacillus malacitensis* (GenBank: AY603656.2), *Bacillus subtilis* subsp. *spizizenii* (GenBank: AF074970.1), *Bacillus subtilis* strain DSM 10 (GenBank: AJ276351.1), *Bacterium* ARb01 (GenBank: KP877501.1), *Bacillus methylotrophicus* strain DB10-1 (GenBank: KU862323.1), *Bacillus tequilensis* strain SBADK5 (GenBank: KF836137.1), *Bacillus vallismortis* (GenBank: AB021198.1), *Bacillus velezensis* strain CR-502 (GenBank: AY603658.1), *Bacillus atrophaeus* (GenBank: AB021181.1), *Bacillus pumilus* strain GR36 (GenBank: KC771051.1), *Bacillus aerius* strain 7PJ-15 (GenBank: KR708841.1), *Bacillus altitudinis* strain JF26 (GenBank: KC172002.1), *Bacillus* sp. strain UK1 (GenBank:KY492395.1), *Bacillus cereus* strain 2000031513 (GenBank: AY138279.1), and *Bacillus coagulans* strain:T5 (GenBank: AB240205.1).

*Bacillus halotolerans* DS5 was identified by 16S rRNA. As shown in Figure 2, phylogenetic tree analysis found that the 16S rRNA nucleotide sequence of this strain was 100% identical to *Bacillus halotolerans* strain ZB201702 (GeneBank: CP029364.1; Zhang et al., 2018).

### Production, purification, and identification of prot DS5

**FIGURE 3:**
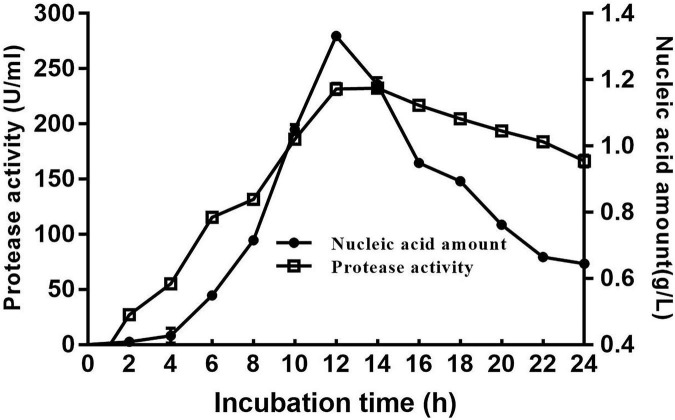
Time course of DS5 cell growth and protease production. Cell growth was detected by measuring the nucleic acid quantity at 260 nm. Each point represents the mean (*n* = 3) ± standard deviation.

The cell growth and protease production of the DS5 strain after culture are shown in Figure 3. The enzyme production started at 4 h, and the enzyme production reached the highest at 12 h in a medium containing 1% dextrin, 0.5% NaCl, 2% soluble starch, and 1% yeast extract (pH 7.0). However, with the extension of fermentation time, the enzyme activity gradually decreased. Therefore, the optimal fermentation time for alkaline protease production was 12 h for *Bacillus halotolerans* DS5.

**FIGURE 4:**
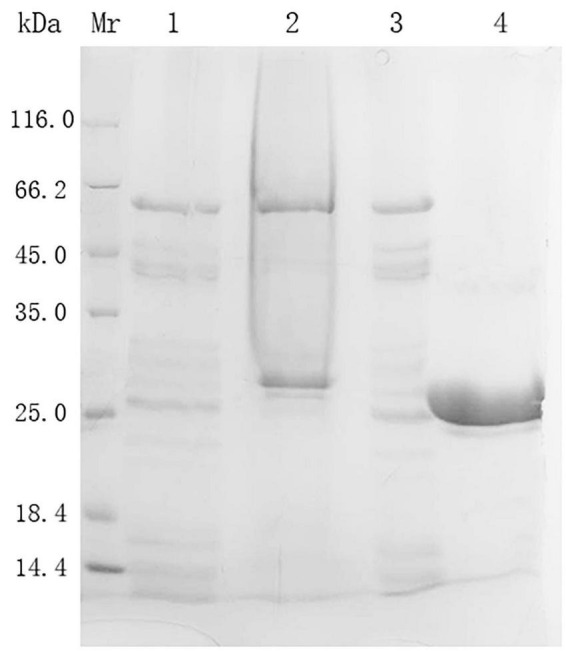
SDS-PAGE analysis of the expression and purification of prot DS5. Mr, protein marker; Lane 1, Cell-free supernatant; Lane 2, 70% (NH4)_2_SO_4_ precipitation sample; Lane 3, Sephadex G-25 desalted sample; Lane 4, sample after Superdex-75 gel chromatography.

The purification data and steps of prot DS5 are summarized in Table 2. After the crude enzyme solution was precipitated by ammonium sulfate and subjected to Superdex-75 gel chromatography, prot DS5 pure enzyme was obtained with a yield of 16% and a purification multiple of 5.6, and a specific activity of 1,501 U/mg. DS5 has successfully achieved homogeneity, and the molecular mass is approximately 27.0 kDa (Figure 4).

**TABLE 2:** Flow sheet for prot DS5 purification from *Bacillus halotolerans* DS5.

Purification step	Total activity (U)	Total protein (mg)	Specific activity (U/mg)	Activity recovery rate (%)	Purification factor (fold)
Cell-free supernatant	74500	279	267	100	1
70% (NH_4_)_2_SO_4_	55875	74	751	75	2.8
Superdex-75	12157	8.1	1501	16	5.6

The prot DS5 protein band was excised from a 12% SDS-PAGE gel and analyzed for protein mass spectra. As described in Supplementary Table 2, six major peptides were obtained by MALDI-TOF mass spectrometry. These peptides share 100% similarity to subtilisin E (39.0 kDa) from *Bacillus subtilis*, indicating that prot DS5 belongs to endopeptidase.

### Enzyme characterization

#### Effect of temperature and pH on the activity and stability of prot DS5

**FIGURE 5:**
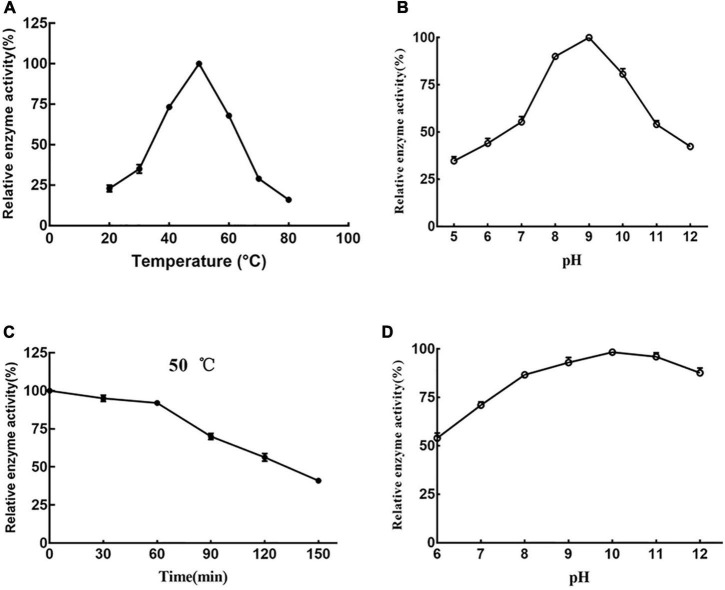
Effects of temperature and pH on prot DS5. Optimum reaction temperature **(A)** and pH **(B)** of the enzyme; **(C)** the stability of the enzyme at 50°C; **(D)** stability of the enzyme at different pH. Each point represents the mean (*n* = 3) ± standard deviation.

Prot DS5 activity was measured at 20–80°C. Prot DS5 had the highest enzyme activity at 50°C (Figure 5A), and the optimal pH is 9.0 (Figure 5B). Prot DS5 was incubated at 50°C for 1 h, the enzyme activity can retain more than 90%, and the half-life is 135 min (Figure 5C). Alkaline proteases in the detergent industry are typically active at around 40–50°C (Mothe and Sultanpuram, 2016).

The prot DS5 was highly stable in the range of pH 7–12 (Figure 5D). We interestingly noticed that the enzyme activity of prot DS5 was basically maintained above 85% after 2.5 h of incubation in the pH range of 8–12. The optimal activity at higher pH and stability for a longer duration at alkaline pH make proteases highly attractive to the detergent industry, since laundry detergents generally operate at a pH of 7–11 (Ramkumar et al., 2018).

#### Effects of metal ions on the activity of prot DS5

The sensitivity of prot DS5 to metal ions was examined. Table 3 revealed that 1 mM of Ba^2+^, Mg^2+^, and Ni^2+^ ions slightly negatively influenced the enzyme activity, and Cu^2+^ severely inhibited the enzyme activity. However, Ca^2+^, Ba^2+^, Mg^2+^, Ni^2+^, Ca^2+^, Fe^2+^, Mn^2+^, and Fe^3+^ ions at 5 mM improved prot DS5 activity, except for Cu^2+^. The most obvious metal ions were 10 mM Ca^2+^ and 15 mM Mn^2+^ ions, which increased the protease activity by 26 and 86.42%, respectively.

**TABLE 3:** Effect of metal ions on the activity of prot DS5.

Agents	Relative activity (%)			
	1 mM	5 mM	10 mM	15 mM
Control	100 ± 6.2	100 ± 5.5	100 ± 5.1	100 ± 5.1
Ca^2+^	104 ± 8.5	115 ± 6.8	126 ± 5.1	120 ± 5.1
Ba^2+^	96 ± 10.7	108 ± 0.1	103 ± 15.3	74 ± 0.6
Mg^2+^	95 ± 2.3	109 ± 5.7	113 ± 4.0	110 ± 4.5
Mn^2+^	118.80 ± 11.2	126.40 ± 5.77	159.48 ± 16.03	186.42 ± 4.89
Ni^2+^	93 ± 3.3	119 ± 2.3	118 ± 2.8	104 ± 0.6
Fe^3+^	102 ± 10.7	102 ± 2.3	114 ± 5.1	110 ± 0.0
Fe^2+^	100 ± 3.4	106 ± 5.66	100 ± 2.3	0 ± 1.1
Cu^2+^	0 ± 17.0	0 ± 33.4	0 ± 0.6	0 ± 4.3

#### Effects of chemical reagents on the activity of prot DS5

As shown in Table 4, non-ionic surfactants (e.g., 1% AEO, 1% Tween-80, 1% Triton X-114, and 1% Tween-20) have almost no effect on the prot DS5. Both anionic surfactant 0.1% SDS and 0.1% cationic surfactant cetyltrimethylammonium bromide (CTAB) have a severe inhibitory effect on enzyme activity. When the concentration is 0.5% for SDS and CTAB, the enzyme activity drops to under 10%. However, the enzyme activity remained above 85.76% at the concentration of 1% H_2_O_2_. In the presence of reducing agents DTT and β-mercaptoethanol, the activity of the enzyme increased or remained basically unchanged, indicating that the enzyme had strong tolerance to oxidants and reducing agents. On the contrary, the protease activity of prot DS5 was inhibited by PMSF and EDTA. Our protease activity was significantly inhibited by PMSF, suggesting that it might be a serine-type protease. The inhibitory effect of EDTA on the enzyme increased with the increase of EDTA concentration. The inhibitory effect of EDTA on the enzyme indicated that the enzyme could obtain the best activity only with the participation of metal ions (like Mn^2+^).

**TABLE 4:** The effect of chemical reagents on the activity of prot DS5.

Agents	Concentration	Relative activity (%)
Control	–	100 ± 6.5
Tween-20	1%	114 ± 1.7
	5%	100 ± 3.2
Tween-80	1%	117 ± 2.0
	5%	97 ± 0.8
TritonX-114	1%	99 ± 2.3
	5%	105 ± 3.0
TritonX-100	1%	150 ± 2.0
	5%	58 ± 0.3
AEO	1%	119 ± 2.1
	5%	106 ± 1.3
H_2_O_2_	1%	85.76 ± 4.45
	5%	64.41 ± 8.35
CTAB	0.1%	64 ± 1.7
	0.5%	16 ± 2.5
SDS	0.1%	76 ± 1.5
	0.5%	9 ± 0.2
PMSF	1 mM	93 ± 3.3
	5 mM	27 ± 0.28
EDTA	1 mM	93.74 ± 8.81
	5 mM	66.02 ± 15.26
DTT	1 mM	117.81 ± 10.65
	5 mM	103.3 ± 18.54
β-mercaptoethanol	1 mM	122.1 ± 21.6
	5 mM	134.54 ± 36.14

#### Salt tolerance analysis of prot DS5

**FIGURE 6:**
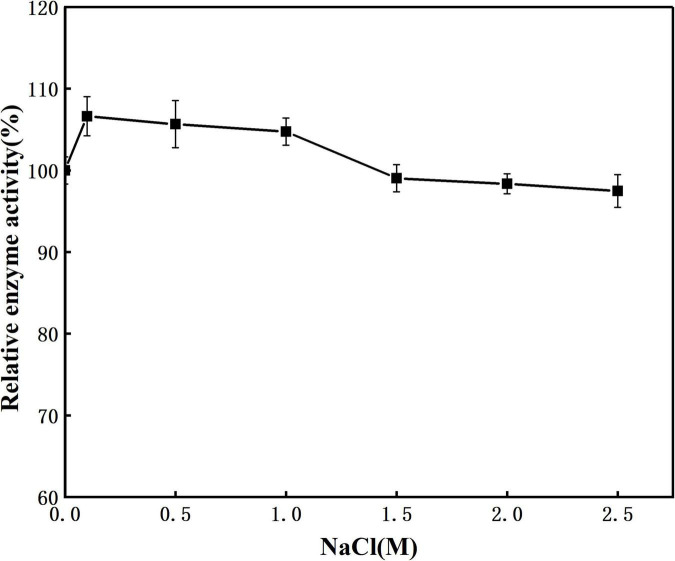
Effect of salinity on the stability of the purified prot DS5.

The effect of NaCl concentration on enzyme activity was shown in Figure 6. When the NaCl concentration was 0–2.5 M, the enzyme activity remained basically unchanged. When the salt concentration reached 2.5 M, prot DS5 maintained a relative enzyme activity of 97.5%. This enzyme may have a protective mechanism corresponding to the high concentration of NaCl environment, so that it can still maintain a high enzyme activity under high salt conditions. The above result indicates that the prot DS5 can keep its properties stable in a high-concentration salt environment and has strong salt tolerance. The salt tolerance mechanism of prot DS5 may be related to secondary structure ratio, salt bridge, and hydrophobicity. Its strong salt tolerance can solve the problem of protease inactivation during the processing of some high-salt foods.

### Prot DS5 substrate specificity

**FIGURE 7:**
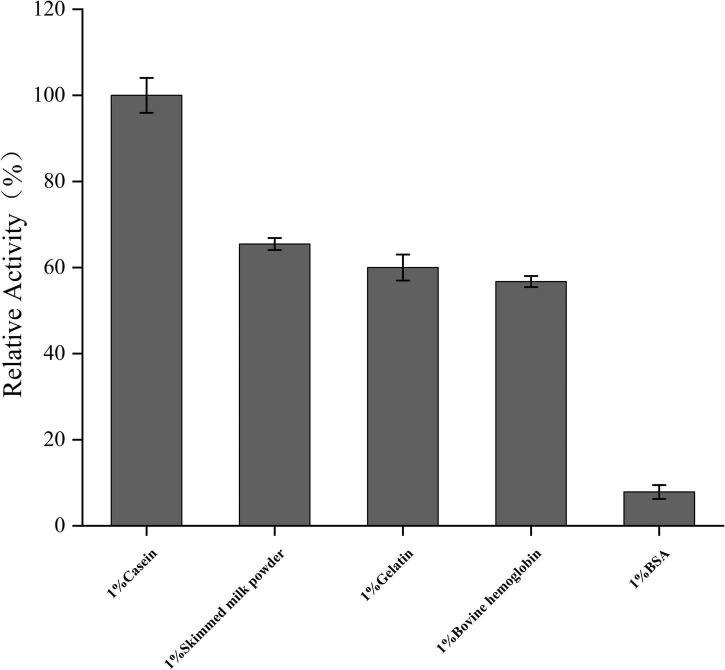
Analysis of substrate-dependent protease activity. Mean values are from three independent experiments.

In all tested substrates (gelatin, casein, skim milk, BSA, and bovine hemoglobin), the enzyme hydrolyzes almost all the above substrates, which also makes it an excellent property for a detergent additive. From the results of substrate specificity detection (Figure 7), it could be seen that the optimal substrate of prot DS5 was casein. Its hydrolysis efficiency is approximately 10-fold that of the substrate BSA.

### Enzyme reaction kinetic parameters

**FIGURE 8:**
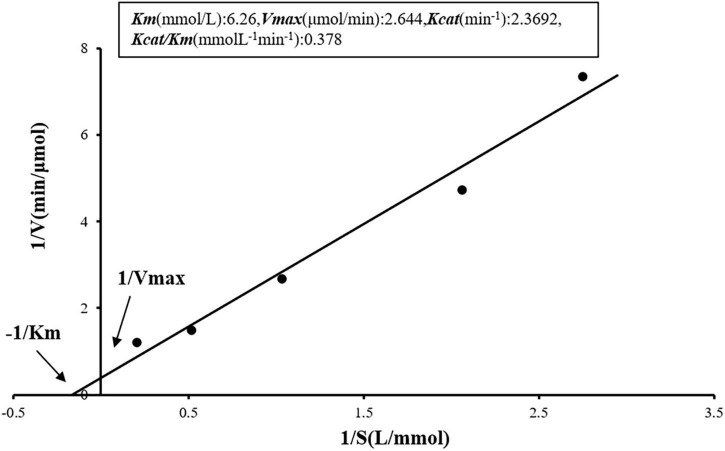
Kinetic analysis of the enzymatic reaction of prot DS5. The enzyme concentration (0.81 mg/ml) was used in the detection. Inset: Kinetic parameters of prot DS5.

The determination of prot DS5 kinetic parameters was studied. Using Lineweaver-Burk plot for visualization, we combined the equations generated from the curve fitting in Figure 8 with the computational rules of the Michaelis-Menten equation kinetics. *V*_*max*_, *K*_*m*_, *K*_*cat*_, and *K*_*cat*_/*K*_*m*_ calculated for the purified enzyme were 2.644 μmol/min, 6.26 mmol/L, 2.3692/min, and 0.378 mmol/L/min, respectively.

### Washing effect of prot DS5

**FIGURE 9:**
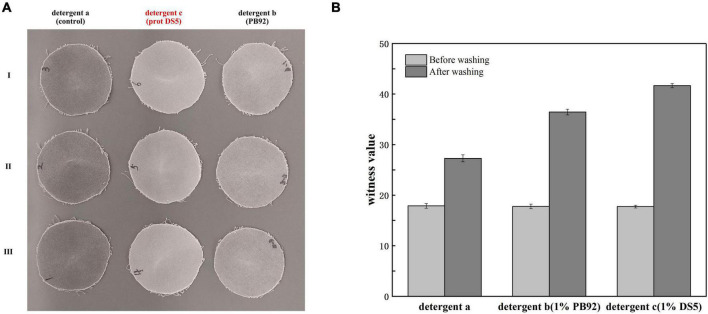
Determination of washing performance of prot DS5. **(A)** Visual appearance evaluation of wash performance. **(B)** Quantitative evaluation of wash performance. Detergent a: 2g Chinese national standard detergent. Detergent b: 2g Chinese national standard detergent + 1% PB92 alkaline protease. Detergent c: 2g Chinese national standard detergent + 1% prot DS5.

By visual appearance evaluation, the addition of prot DS5 showed better stain removal ability compared with control or PB92 protein test cloth (Figure 9A). The visual appearance of better cleaning was supported by quantitative evaluation, and we compared whiteness values to evaluate detergent performance. Specifically, using the calculation method of the value of detergency (R) in GB/T13174-2008 (China National Standard), the R of detergents a, b, and c was 9.50, 18.68, and 23.93, respectively (Figure 9B). The results show that the washing performance of detergents b and c was higher than a. In conclusion, prot DS5 had an excellent stain removal effect.

## Discussion

In soil, microorganisms (Singh et al., 2011), animal excreta (Sánchez-Ramos et al., 2004), plants (Milton and Chawla, 1986), and dry-wet deposition (Rudenskaya and Pupov, 2008) constitute the main sources of alkaline proteases. Bacillus (Zhang et al., 2022), Actinomyces (Espersen et al., 2021), and Fungi (Damare et al., 2020) have been reported to produce alkaline protease. Among them, Bacillus is widely used in commercial to produce alkaline protease, such as *Bacillus licheniformis* used by NovoNordisk, a Danish enzyme manufacturer. A new Bacillus producing alkaline protease was found in the soil of mountain areas in Shanxi Province of China. It was identified as *Bacillus halotolerans* by 16S rRNA and named as *Bacillus halotolerans* DS5.

In general, the fermentation time of Bacillus producing alkaline protease is about 24 h–72 h. In previous studies, *Bacillus* B001 reached the highest enzyme activity when cultured for 30 h (Deng et al., 2010). *Bacillus* sp. APP1 (Chu, 2007), *Bacillus aquimaris* VITP4 strain (Thaz and Jayaraman, 2014), *Bacillus licheniformis* strain K7A (Hadjidj et al., 2018), *Bacillus* sp. ATCC 6633 (Dias et al., 2008), and endophytic *Bacillus halotolerans* strain CT2 (Dorra et al., 2018) reached the maximum protease production after 48 h incubation. The cell growth and protease production reached the highest value at 22 h for *Bacillus safensis* strain RH12 (Rekik et al., 2019). But for *Bacillus safensis* S406 (Mhamdi et al., 2017), *Bacillus* sp. SM2014 (Jain et al., 2012), *Bacillus* sp.DEM05 (Mohamadi et al., 2021), *Bacillus pumilus* ATCC7061 (Gomaa, 2013), and *Bacillus pumilus* SG2 (Sangeetha et al., 2011), the protease production reached the highest value at 72 h. Compared with these strains, DS5 strain has the greatest advantage of short fermentation time. The maximum output of protease can be reached after 12 h, which greatly shortens the fermentation time and saves the cost. With the continuous development of science and technology, people have made enormous progress in the field of industrial enzyme research, and a large flow of data on bacterial proteolytic enzymes is emerging, but there is still less report on the separation, purification, and identification of protease from *Bacillus halotolerans* (Dorra et al., 2018). In view of the limited information about this enzyme from *Bacillus halotolerans*, it is very valuable to purify and characterize it from a newly isolated bacterium.

Alkaline protease in the detergent industry usually has activity at about 40–50°C (Mothe and Sultanpuram, 2016). The optimum temperature of Savinase is 50–60°C (Beg and Gupta, 2003). The optimum temperature of prot DS5 was 50°C, and 90% of the initial activity was still observed after 60-min incubation at the same temperature. This result is consistent with the report from endophytic *Bacillus halotolerans* strain CT2 (50°C; Dorra et al., 2018), *haloalkaliphilic Bacillus lehensis* JO-26 (50°C; Bhatt and Singh, 2020), *Geobacillus steelothermophilus* (50°C; Chang et al., 2021), and *Bacillus* sp. DEM05 (50°C; Mohamadi et al., 2021). Prot DS5 has wide pH tolerance and high activity within 6–12. To be the standard for detergents, the enzyme should first be resistant to alkaline conditions (Jain et al., 2012). The optimum pH value of prot DS5 is 9.0. This result is consistent with the report from endophytic *Bacillus halotolerans* strain CT2 (Dorra et al., 2018), *Bacillus safensis* strain RH12 (Rekik et al., 2019), and *Bacillus invictae* AH1 strain (Hammami et al., 2020). These findings indicate that it meets the protease properties required by the detergent industry. To be the first choice of detergent, alkaline protease should have the ability to hydrolyze a variety of proteins. Casein is the best substrate for prot DS5 relative to other substrates. It is consistent with the optimal substrate for protease from *Bacillus megaterium*-TK1 (Manavalan et al., 2020) and *Caldicoprobacter guelmensis* (Bouacem et al., 2015), *Geotrichum candidum* QAUGC01 (Muhammad et al., 2019), and *Geobacillus steelothermophilus* (Chang et al., 2021). Prot DS5 can remove various protein stains, and the potential of these enzymes to prepare hydrolysates in some industries can also be explored (Tu et al., 2018).

As cofactors of enzymes, metal ions have obvious activating or inhibiting effects on proteases. Many researchers have reported that alkaline proteases require metal ions, such as Ca^2+^, Zn^2+^, and Fe^2+^ to maintain their stable properties at higher temperatures (Bouacem et al., 2015; Ibrahim et al., 2019). Ca^2+^ and other divalent ions, such as Mn^2+^ and Mg^2+^, are known to enhance protease activity. In our research, we found that Mg^2+^ and Ca^2+^ enhanced the activity of prot DS5, which further indicated that prot DS5 is suitable for washing in hard water (presence of Mg^2+^ and Ca^2+^). With the continuous increase of Mn^2+^ concentration, the activity of prot DS5 showed a gradually increasing trend. When the Mn^2+^ concentration reached 15 mM, the activity of prot DS5 increased to 186.42%. Mn^2+^ ions have been reported to activate proteases from organisms, such as endophytic *Bacillus halotolerans* strain CT2 (Dorra et al., 2018), *Bacillus circulans* MTCC 7942 (Patil et al., 2016), *Bacillus pumilus* TMS55 (Ibrahim et al., 2011), *Stenotrophomonas maltophilia* (MTCC 7528; Kuddus and Ramteke, 2009), *Bacillus* sp. ZJ1502 (Yu et al., 2019), and *Bacillus amyloliquefaciens* strain HM48 (Mushtaq et al., 2021). However, the exact mechanism of Mn^2+^ remains to be further elaborated. Hydrogen peroxide is a strong oxidant, and most enzymes will be inactivated within 1–2 min in the presence of hydrogen peroxide (Yu et al., 2019). Prot DS5 remained highly active (64.41%) in 5% H_2_O_2_. In addition, DTT and β-mercaptoethanol slightly activated prot DS5. Prot DS5 also keeps highly active (106.15%) in high salinity solution (2.5M). Studies on the effects of different surfactants have shown that non-ionic surfactants (Tween-80, Tween-20, AEO, and Triton X-114) slightly increased prot DS5 protease action. The alkaline protease of endophytic *Bacillus halotolerans* CT2 also has a similar effect (Dorra et al., 2018). Taken together, our results demonstrate that the resistance to salt, oxidants, reductants, surfactants, and metal ions of prot DS5 is better than those of prot CT2.

As an essential component in detergent, protease must be soluble with most detergent additives, including bleach, surfactant, oxidant, and other additives (Sharma et al., 2021). In recent years, researchers have screened alkaline proteases with their own characteristics from different new strains; however, the stability of most new alkaline proteases is reflected in one or several aspects. For example, some proteases have high resistance to NaCl, but poor resistance to oxidants and reductants. Protease from *halotolerant alkaliphilic Bacillus* sp. NPST-AK15 (Ibrahim et al., 2015) is highly stable in NaCl up to 20% (w/v) and slightly activated by hydrogen peroxide, but the reductants β-mercaptoethanol and DTT significantly inhibited the enzyme activity. In the presence of 10 mM Ca^2+^ and Tween-80, the enzyme activity of an alkaline protease from fermented bean curd (Yu et al., 2019) can be increased by 21 and 30%, while its tolerance to H_2_O_2_ is weak. The alkaline protease produced by *Bacillus subtilis* DR8806 (Farhadian et al., 2015) can improve the enzyme activity in the presence of Mg^2+^, Ca^2+^, K^+^, and Fe^2+^, but the temperature, pH stability, and tolerance to hydrogen peroxide are poor. Thus, compared with the reported Bacillus surfactant and detergent stabilizing enzyme, the prot DS5 was found to have better activity and stability, indicating that it has more application value, which may pave the way for its development in industry.

## Conclusion

We proposed a novel alkaline protease derived from a novel *Bacillus halotolerans* strain. The extracellular protease (prot DS5) was also purified and characterized. Prot DS5 exhibits higher activity and stability at 50°C and a wider pH range. Prot DS5 not only is salt tolerant but also shows good tolerance to oxidants and reducing agents, which are the basic conditions for detergent enzymes. DS5 has a very amazing detergency ability, which can be used as a candidate enzyme for industrial applications. It needs further investigation into the structure-function relationship of the enzyme by genetic engineering and protein engineering.

## Data availability statement

The original contributions presented in this study are included in the article/Supplementary material, further inquiries can be directed to the corresponding author.

## Author contributions

YW and YS conceived and designed the study. YW, JQ, GZ, and XZ performed the experiments. YW performed the data analyses and wrote the manuscript. LW and YS participated in modifying the manuscript. All authors contributed to the article and approved the submitted version.
