# Supplementary material for: Characterization of redox and salinity-tolerant alkaline protease from Bacillus halotolerans strain DS5
Source: Front Microbiol. 2022 Aug 18;13:935072. doi: 10.3389/fmicb.2022.935072 (PMC9434114; doi:10.3389/fmicb.2022.935072)
Supplement: Supplementary file 1 [file Data_Sheet_1.docx]

Supplementary Material

Characterization of Redox and Salinity-tolerant alkaline protease from *Bacillus halotolerans* strain DS5

YangxuanWen^1†^, JiyuQiang^1†^, GuixuZhou^1^, XiaoboZhang^1^, Lei Wang^1^, YaweiShi^1*^

^1^Key Laboratory of Chemical Biology and Molecular Engineering of Ministry of Education, Institute of Biotechnology, Shanxi University, Taiyuan, China

*** Correspondence:**

Yawei Shi

yaweishi@sxu.edu.cn

†These authors have contributed equally to this work

**Table Captions**

**Table S1** Screening and rescreening of protease producing strain.

**Table S2** Partial sequence fragments of protease in *Bacillus halotolerans* DS5 identified by MALDI-TOF MS.

1. **Tables**

**Table S1** Screening and rescreening of protease producing strain

| **Strain Numbering** | **Transparent circle diameter**  **(D/mm) ^a^** | **Colony diameter**  **(d/mm) ^a^** | **Transparent circle diameter**  **/Colony diameter**  **(D/d) ^a^** | **Enzyme activity**  **(U/mL) ^a,b^** |
| --- | --- | --- | --- | --- |
| DS5 | 5.73±0.02 | 3.45±0.01 | 1.66 | 85.09±4.61 |
| DS11 | 4.12±0.01 | 3.03±0.01 | 1.36 | 51.46±0.58 |
| AS4 | 6.13±0.01 | 3.12±0.01 | 1.97 | 37.63±0.67 |
| Note: ^a^The experiment was carried out triplicates, and the data are represented as the mean±standard deviation  ^b^The protease enzyme activity was measured using the Folin phenol color method | | | | |

**Table S2** Partial sequence fragments of protease in *Bacillus halotolerans* DS5 identified by MALDI-TOF MS

| **Proteases** | **Positioning of peptides detected by MALDI-TOF MS** | **Sequence coverage**  **(%)** |
| --- | --- | --- |
| Subtilisin E (*Bacillus Subtilis*) | **1** MRSKKLWISL LFALTLIFTM AFSNMSAQAA GKSSTEKKYI VGFKQTMSAM  **51** SSAKKKDVIS EKGGKVQKQF KYVNAAAATL DEKAVKELKK DPSVAYVEED  **101** HIAHEYAQSV PYGISQIK**AP ALHSQGYTGS NVKVAVIDSG IDSSHPDLNV**  **151 RGGASFVPSE** **TNPYQDGSSH** **GTHVAGTIAA** **LNNSIGVLGV** **APSASLYAVK**  **201 VLDSTGSGQY** **SWIINGIEWA** **ISNNMDVINM** **SLGGPTGSTA** **LK**TVVDKAVS  **251** SGIVVAAAAG NEGSSGSTST VGYPAK**YPST** **IAVGAVNSSN** **QRASFSSAGS**  **301 ELDVMAPGVS** **IQSTLPGGTY** **GAYNGTSMAT** **PHVAGAAALI** **LSKHPTWTNA**  **351 QVR**DRLESTA TYLGNSFYYG K**GLINVQAAA Q** | **55** |
